# Estimating relationship between the time over threshold and energy loss by photons in plastic scintillators used in the J-PET scanner

**DOI:** 10.1186/s40658-020-00306-x

**Published:** 2020-06-05

**Authors:** S. Sharma, J. Chhokar, C. Curceanu, E. Czerwiński, M. Dadgar, K. Dulski, J. Gajewski, A. Gajos, M. Gorgol, N. Gupta-Sharma, R. Del Grande, B.C. Hiesmayr, B. Jasińska, K. Kacprzak, Ł. Kapłon, H. Karimi, D. Kisielewska, K. Klimaszewski, G. Korcyl, P. Kowalski, T. Kozik, N. Krawczyk, W. Krzemień, E. Kubicz, M. Mohammed, Sz. Niedzwiecki, M. Pałka, M. Pawlik-Niedźwiecka, L. Raczyński, J. Raj, A. Ruciński, S. Shivani, R.Y. Shopa, M. Silarski, M. Skurzok, E.Ł. Stępień, W. Wiślicki, B. Zgardzińska, P. Moskal

**Affiliations:** 1 Faculty of Physics, Astronomy and Applied Computer Science, Jagiellonian University, prof. Stanisława Łojasiewicza 11, Cracow, 30-348 Poland; 2 INFN, Laboratori Nazionali di Frascati, Frascati, 00044 Italy; 3 Institute of Nuclear Physics PAN, Cracow, Poland; 4 Institute of Physics, Maria Curie-Skł odowska University, Lublin, 20-031 Poland; 5 Faculty of Physics, University of Vienna, Vienna, 1090 Austria; 6 Department of Complex Systems, National Centre for Nuclear Research, Otwock-Świerk, 05-400 Poland; 7 High Energy Physics Division, National Centre for Nuclear Research, Otwock-Świerk, 05-400 Poland; 8 2nd Department of General Surgery, Jagiellonian University Medical College, Cracow, Poland; 9 Department of Physics, College of Education for Pure Sciences, University of Mosul, Mosul, Iraq

**Keywords:** Positron emission tomography, Time over threshold, Positronium atoms, Medical imaging

## Abstract

**Purpose:**

The time-over-threshold (TOT) technique is being used widely due to itsimplications in developing the multi-channel readouts, mainly when fast signal processing is required. Using the TOT technique, as a measure of energy loss instead of charge integration methods, significantly reduces the signal readout costs by combining the time and energy information. Therefore, this approach can potentially be utilized in J-PET tomograph which is built from plastic scintillators characterized by fast light signals. The drawback in adopting this technique lies in the non-linear correlation between input energy loss and TOT of the signal. The main motivation behind this work is to develop the relationship between TOT and energy loss and validate it by the J-PET tomograph setup.

**Methods:**

The experiment was performed using a ^22^Na beta emitter source placed in the center of the J-PET tomograph. This isotope produces photons of two different energies: 511 keV photons from the positron annihilation (direct annihilation or through the formation of a para-positronium atom or pick-off process of ortho-positronium atoms) and a 1275 keV prompt photon. This allows the study of the correlation between TOT values and energy loss for energy ranges up to 1000 keV. Since the photon interacts predominantly via Compton scattering inside the plastic scintillator, there is no direct information of the energy deposition. However, using the J-PET geometry, one can measure the scattering angle of the interacting photon. Since the ^22^Na source emits photons of two different energies, it is necessary to know unambiguously the energy of incident photons and their corresponding scattering angles in order to estimate energy deposition. In summary, this work presents a dedicated algorithm developed to tag photons of different energies and studying their scattering angles to calculate the energy deposition by the interacting photons.

**Results:**

A new method was elaborated to measure the energy loss by photons interacting with plastic scintillators used in the J-PET tomograph. We find the relationship between the energy loss and TOT is non-linear and can be described by the functions TOT = A0 + A1 * ln(E _*dep*_ + A2) + A3 * (ln(E _*dep*_ + A2))^2^ and TOT = A0 - A1 * A2$^{E_{dep}}\phantom {\dot {i}\!}$. In addition, we also introduced a theoretical model to calculate the TOT as a function of energy loss in plastic scintillators.

**Conclusions:**

A relationship between TOT and energy loss by photons interacting inside the plastic scintillators used in J-PET scanner is established for a deposited energy range of 100–1000 keV.

## Background

The time-over-threshold (TOT) technique was first time introduced by Nygren and Millaud [1] and proved to be an excellent solution for multi-channel readouts [2]. In the TOT method, one measures the signal pulse width at a selected threshold, which can be used to estimate the signal’s charge. For energy deposition estimation, this method is less precise in comparison to the charge integration method. However, it reduces the readout costs by using only the time to digital converter (TDC), herewith combining both timing and energy information. The application of the TOT method for the energy loss determination may be of particular advantage in the newly developed Jagiellonian-positron emission tomograph (J-PET) [3, 4] which is based on plastic scintillators characterized by fast light signals with rise and decay times of the order of ≈ 1 ns [5, 6] and thus being about two orders of magnitude shorter than signals from crystals used in the current PET devices [7–9]. Therefore, the application of the TOT method for PET tomographs built from plastic scintillators will enable fast signal processing and significantly reducing signal acquisition dead time with respect to the crystal based tomographs. Despite many advantages like compactness of signals readout and low power consumption, the TOT technique confronts the challenge in terms of non-linear input energy to pulse width conversion [10–12]. It has been reported that using multiple fixed triggering thresholds [13, 14] or dynamic threshold levels [15, 16] for estimating the TOT values alleviates the problem of non-linearity to a significant extent.

In PET applications, the precise determination of the photon’s interaction position, hit time, and energy loss is crucial. Currently, in PET scanners, crystal scintillators are used and they enable the determination of the energy of the interacting photons as they undergo photoelectric effect [17–19]. In contrast to crystals in plastic scintillators, the incident photon interacts via Compton scattering depositing only part of its energy. Thus, there is no direct information on the energy deposition of the interacting photon.

TOT studies with plastic scintillators are very scarce [20]. In the recent work of Ashrafi and Gol [21], the energy calibration of a plastic scintillator based on Compton scattering and observing the light yield by using various monochromatic photon sources was reported. In this work, we take advantage of the multilayer cylindrical acceptance of the J-PET scanner [3, 22] which allows us to determine the direction of the photon before and after the scattering which in turn gives us access to its scattering angle [4, 23]. Thus, knowing the incident energy and the measured scattering angle of the initial photon, the deposited energy in the photon’s interaction can be calculated [24].

For the present study, a ^22^Na source was used which emits photons of two different energies: 511 keV (annihilation) and 1275 keV (prompt). The 511 keV photons originate from the direct electron-positron annihilation, from the decay of para-positronium, and from the annihilation of ortho-positronium atoms via the pick-off processes [25–27]. Since the aim of the study is to establish the relationship between TOT and energy loss, we need to develop an algorithm capable to clearly distinguish between the annihilation and prompt photons as well as a method of proper correspondence of the measured signals to the initial and scattered photons. To achieve this, we performed studies based on the assumptions that 511 keV photons emitted in the annihilation of e ^+^*e*^−^ are always back-to-back due to momentum conservation. Secondly, to identify the prompt photons, we take advantage of the decay of long-lived ortho-positronium atoms [28] which in the XAD −4 porous polymer [29] on the average decay about 90 ns after the emission of a prompt photon. In this article, the presented algorithm allows us for establishing a relationship between TOT and energy deposition in plastic scintillators, a step further towards medical imaging with low cost PET devices.

## Methods

**Fig. 1:**
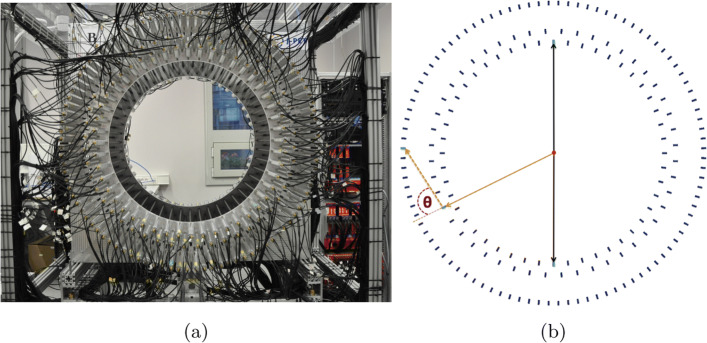
**a** The photo of J-PET scanner with the front view (see text for description). **b** The cross section of J-PET with superimposed arrows depicting the interaction of 511 keV photons (black solid arrows) originating from positron-electron annihilation and prompt photon 1275 keV before (orange solid arrow) and after (orange dashed arrow) scattering inside the scintillator. Red dot in the center represents the ^22^Na source

**Fig. 2:**
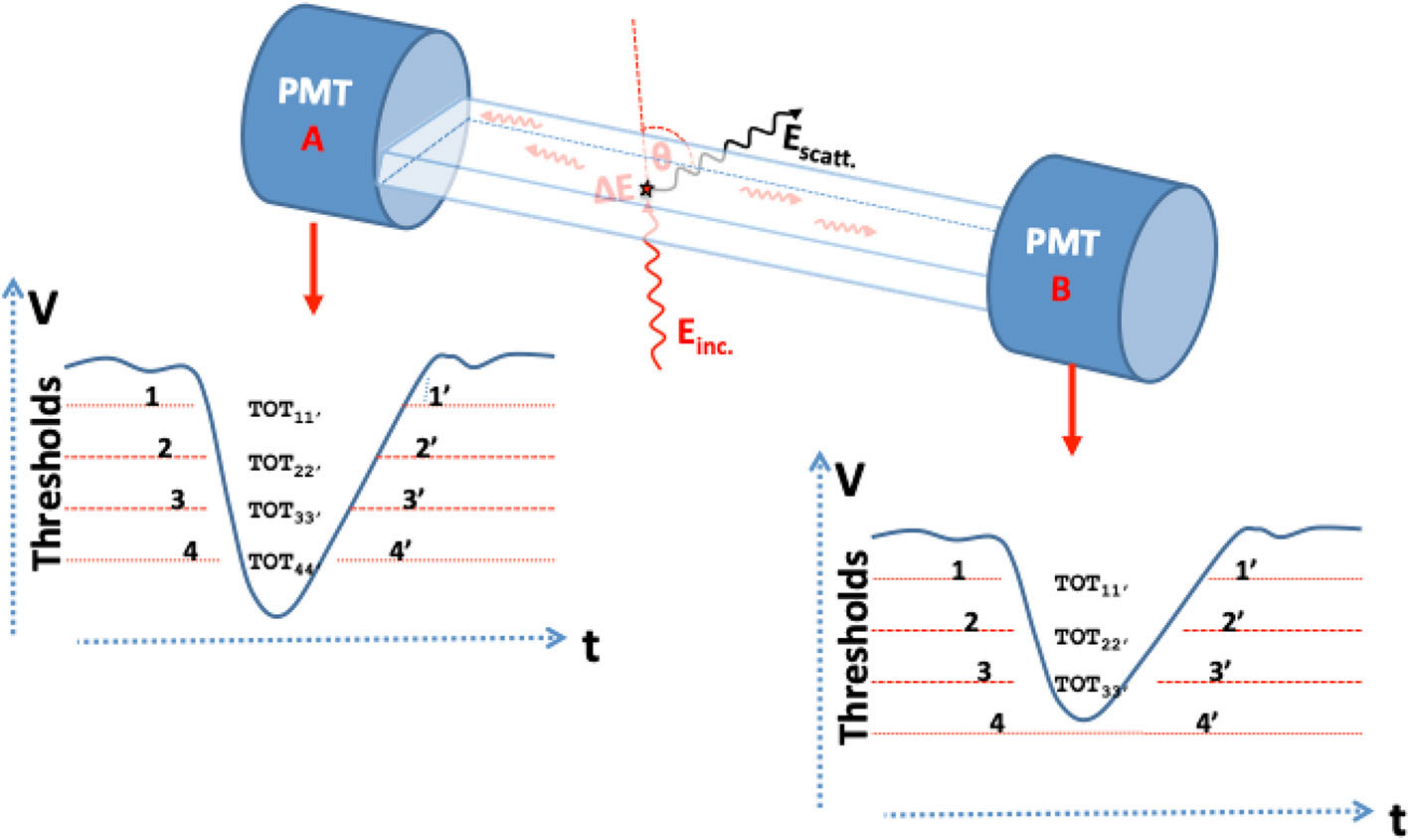
Illustration of the analog signals obtained at photomultipliers as the outcome of the energy deposited by the photon in the interaction with plastic scintillator. Each signal is probed at four fixed thresholds. The TOT value is calculated at each pre-defined voltage threshold, and the sum of all four TOT values from both photomultipliers (PMTs) gives the final TOT value, Eq. 1

The Jagiellonian-positron emission tomograph (J-PET) consists currently of 192 plastic scintillators made of EJ-230 material. A single detection module is composed of a scintillator with dimension 0.7 × 1.9 × 50 cm^3^ connected with a R9800 Hamamatsu photomultiplier on each side [30, 31]. The detection modules are axially arranged in three layers: the first and second layer are composed of 48 modules whereas the third layer is the arrangement of 96 modules. Layers are not overlaying and have a diameter of 85 cm, 93.5 cm, and 115 cm, respectively. The front view of three layer prototype of the J-PET scanner is shown in Fig. 1a whereas Fig. 1b exhibits its cross section. A Multi-Voltage Threshold mezzanine (MVT) board is used to probe signals at four fixed thresholds (80, 160, 240, and 320 mV) in the voltage domain (within the accuracy of 20 ps RMS) [32] to achieve the good resolution of determining the hit time and place of interaction of photon inside the scintillator [3, 31, 33–35]. Using MVT boards connected to readouts allows us to exploit the FPGAs differential inputs as comparators. The signals from the MVT boards are sampled using TDCs implemented in the FPGA devices [36–39] whereas in standard approach, external comparator chips are used additionally [40, 41]. The data is stored in a trigger less mode with an ability to handle the data stream with rates of about 8 Gbps [42, 43]. The position of interaction along the plastic strip is calculated based on the time difference of light signals arriving at both photomultipliers. The hit-time resolution for the registration of 511 keV photon is ≈ 155 ps [30] whereas the spatial resolution on xy is 4–5 mm and on *z*-axis is ≈ 25 mm [44]. Signals from the plastic scintillators are very fast (rise time ≈ 0.5 ns, fall time ≈ 1.8 ns) [6] and are prone to much lower pile-ups with respect to crystal based detectors with an order of magnitude larger fall times [45]. Therefore, in order to avoid additional dead times in the detection system, instead of direct charge measurement only timing of the signal is used. This allows us to handle higher rates of data collection. The photon’s interaction in the scintillator and the arrival time of the signals in photomultipliers at each end is measured. The TOT approach is adopted instead of the charge integration method. In TOT approach, the time difference between the leading and trailing edge of signal pulse crossing the applied thresholds is measured. The schematic presentation is shown in Fig. 2. The total TOT, used as a measure of the energy deposition, is estimated as a sum of TOT values measured for all thresholds at both sides of scintillator (Eq. 1):

1 $$ \hspace{2.5cm} {TOT} = \sum_{PMT = A,B} \sum_{{Thr}_{1-4}}^{{TOT}_{PMT,Thr}}  $$

where *TOT*_*PMT*,*Thr*_ is representing the sum of TOT values over all four threshold on the signals measured from both photomultipliers of each scintillator. The estimation of the charge collection using the pulse width of the signal is less precise and suffers a strongly non-linear relationship between TOT and energy deposition. The plastic scintillators are composed of hydrocarbons with low atomic (*Z*) number; consequently, gamma photons interact in the plastic scintillators predominantly through Compton scattering and deposit only part of their energy. The information of the deposited energy is important to reduce the scatter fraction by requiring that the energy loss is larger than 200 keV [3, 23, 34]. To process the measured data, a dedicated framework for offline data analysis was used [46, 47].

### Experimental set-up

**Fig. 3:**
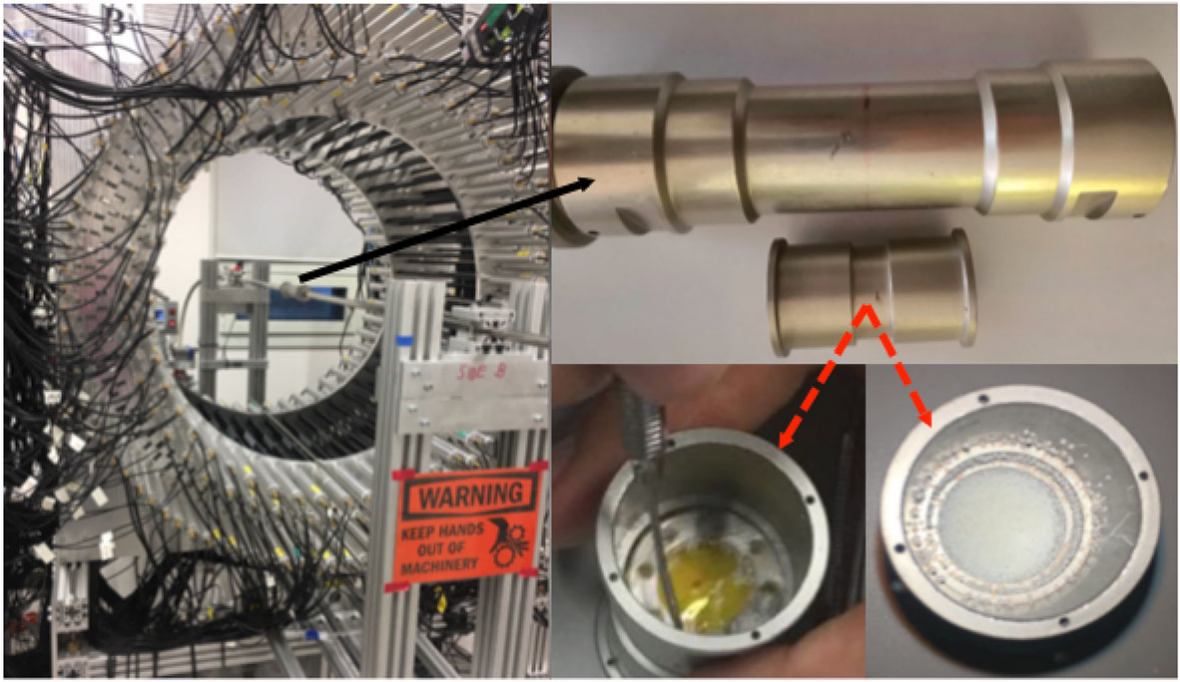
Experimental set-up of the annihilation chamber with the source placed at the center of the J-PET tomograph

**Fig. 4:**
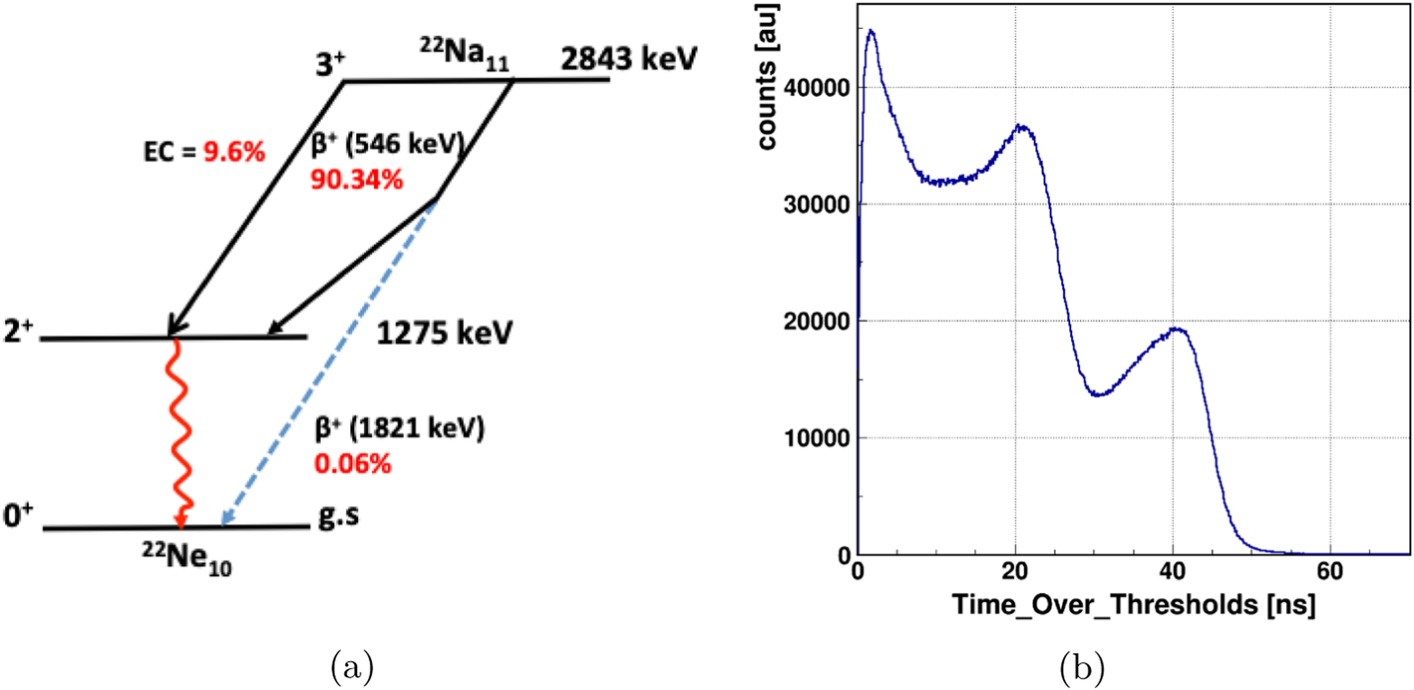
**a** The decay scheme of the ^22^Na source. **b** The typical TOT spectrum obtained using the ^22^Na source

The measurements were performed with a ^22^Na source (1 MBq activity) wrapped inside a very thin Kapton foil. The experimental set-up is shown in Fig. 3. The left panel shows a picture of the J-PET tomograph with the placement of a barrel shape source holder of length ≈14 cm and diameter ∼ 3.16 cm (at the center). The source surrounded by the porous material was put inside a small chamber of thin layer made of aluminum and placed at the center of the holder (see the upper inset of right panel in Fig. 3). The placement of the source covered with porous material is shown at the lower right insets. In this experiment, the XAD-4 polymer was used which increases the probability of formation of positronium (Ps) atoms [29]. Using the ^22^Na source gives a possibility to estimate the lifetime of the Ps atom by registering the annihilation photons and prompt gamma which is a feature that will allow a novel application in medical imaging. J-PET is optimized to register multiple photons simultaneously, and thus, it can be used to reconstruct the image of average lifetime of the ortho-positronium (o-Ps → 3 *γ*) which is formed in patient body during the PET scanning process. The average lifetime of o-Ps strongly depends on the free volume between atoms and thus can be used as a diagnostic indicators of human tissues [26, 27]. In the decay of ^22^Na source (Fig. 4a), the positron is emitted leaving behind an excited state of ^22^Ne nucleus which eventually de-excites via the emission of a prompt photon on average within the time interval of ≈ 3.7 ps. The time difference between the interaction of annihilation and prompt photons is then with a very good approximation equal to lifetime of Ps atom [26]. Gamma photons mainly interact inside the plastic scintillator via Compton scattering.

**Fig. 5:**
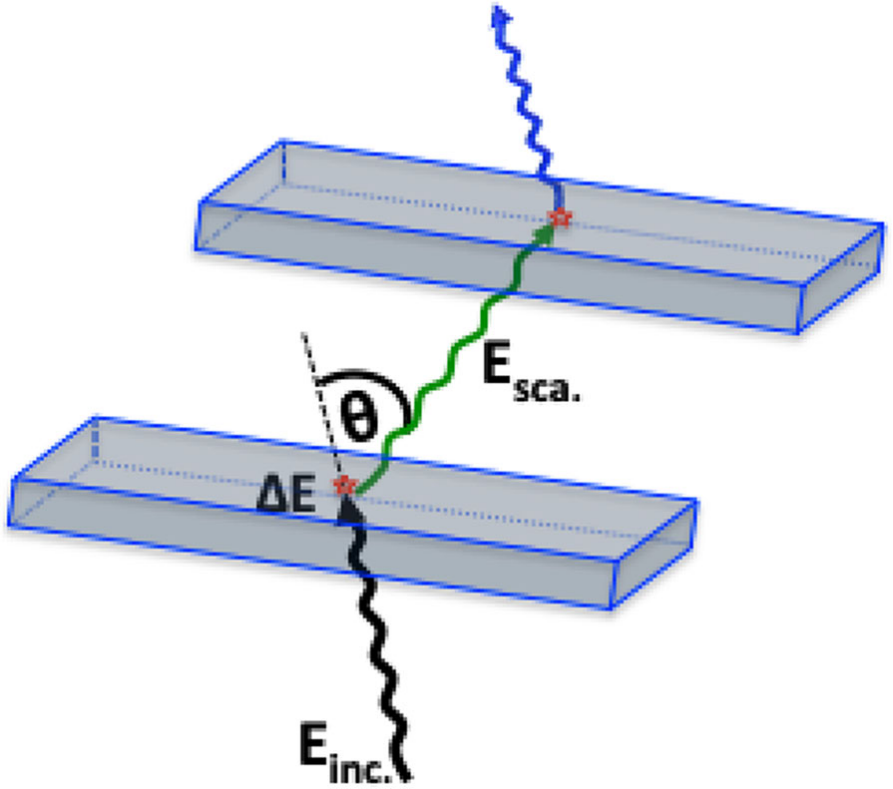
The incoming and scattered photon interactions are presented with two exemplary scintillators. Knowing the positions of emission of *E*_*inc*_ photon and positions of interactions of *E*_*inc*_ and *E*_*sca*_ photons inside the scintillators allow to estimate the scattering angle

Figure 4b shows the TOT spectrum for all registered photons. The clear Compton edges are visible at about 22 ns and 46 ns corresponding to 511 keV and 1275 keV photons, respectively. The observed enhancement of contributions for the lower values of TOT (below 10 ns) is from the interaction of scattered photon and photons originating from the 3-photon decay of o-Ps atoms. It is worth to emphasize that TOT values could be used to disentangle between the annihilation and prompt photons to some extent. However, in the overlapping region for TOT values below 30 ns, the identification would be ambiguous. Therefore, we developed a dedicated algorithm to uniquely identify the photons of energies 511 keV and 1275 keV irrespective of the TOT values (described in the “Events selection” section). Furthermore, the registration of initial and scattered photons allows us to determine the scattering angle *θ* (see Fig. 5). Knowing the energy (*E*_*inc*_) and scattering angle (*θ*) of the initial photon give access to energy deposition *Δ*E inside the scintillator [24]:

2 $$ \hspace{2.5cm} \Delta{E}=E_{\text{inc}}\left [ 1-\frac{1}{1+\frac{E_{\text{inc}}}{511~keV}\left (1-\cos\theta \right)} \right ]   $$

Determination of *Δ*E and the measurement of TOT enables to establish the relation between these two variables.

In this work, events with three interactions were studied, assuring that all three hits forming the events are occurring in distinct scintillators. Furthermore, a photon interaction inside the scintillator is termed as hit. The required information (E _*inc*_,*θ*) can be extracted based on two hits only. However, the third hit allows event categorization in a way that we can differentiate the photons of different energies and conjecture their origins.

### Events selection

**Fig. 6:**
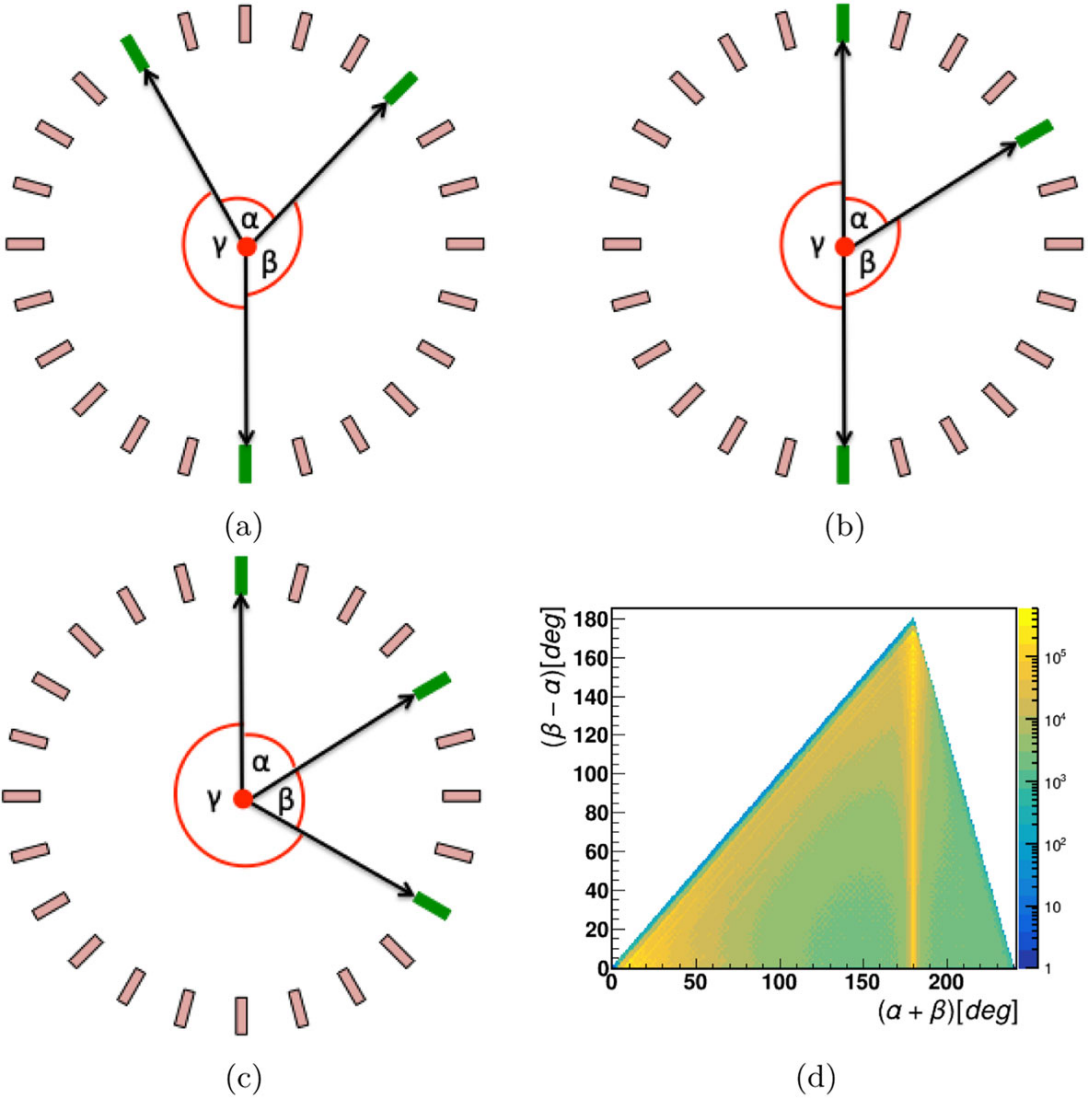
**a**–**c** The three cases based on the angular correlation between the 3 hits in an event. The origin of the visualized events is explained in the text. **a** The case when the sum of two smallest angles is greater than 180^∘^. **b** The case when sum is 180^∘^. **c** When the sum of two smallest angles is less than 180^∘^. **d** The measured scatter plot of the difference (*β*−*α*) vs the sum (*α*+*β*) of the two smallest angles

In this analysis, we first select the events containing three hits only. The angular correlations between the registered photons in three hit events can be used to partially identify the origin and energy of photons [48, 49]. A pictorial view of the three hits (green rectangles) and their angular correlations are presented in Fig. 6a–c. For clarity, only a few scintillators mimicking one layer of J-PET scanner are shown. The angles between three hits ordered from the smallest to the largest are *α*, *β*, and *γ*, respectively. The measured sum of the two smallest angles vs their difference is shown in Fig. 6d which shows three distinct regions: (i) (*α* + *β*) >180^∘^, electron-positron annihilation into three photons originating from direct *e*^+^*e*^−^ annihilation or from the decay of o-Ps atoms; (ii) (*α* + *β*) = 180^∘^, two back-to-back photons originating either from direct *e*^+^*e*^−^ annihilation or from the decay of p-Ps atom (singlet state of Ps) or from the pick-off of o-Ps; (iii) (*α* + *β*) <180^∘^, one or two of the hits are from scattering of initial photon or prompt photon. The coincidence time window for an event was fixed at 200 ns in the analysis.

### Identification of 511 keV photons

**Fig. 7:**
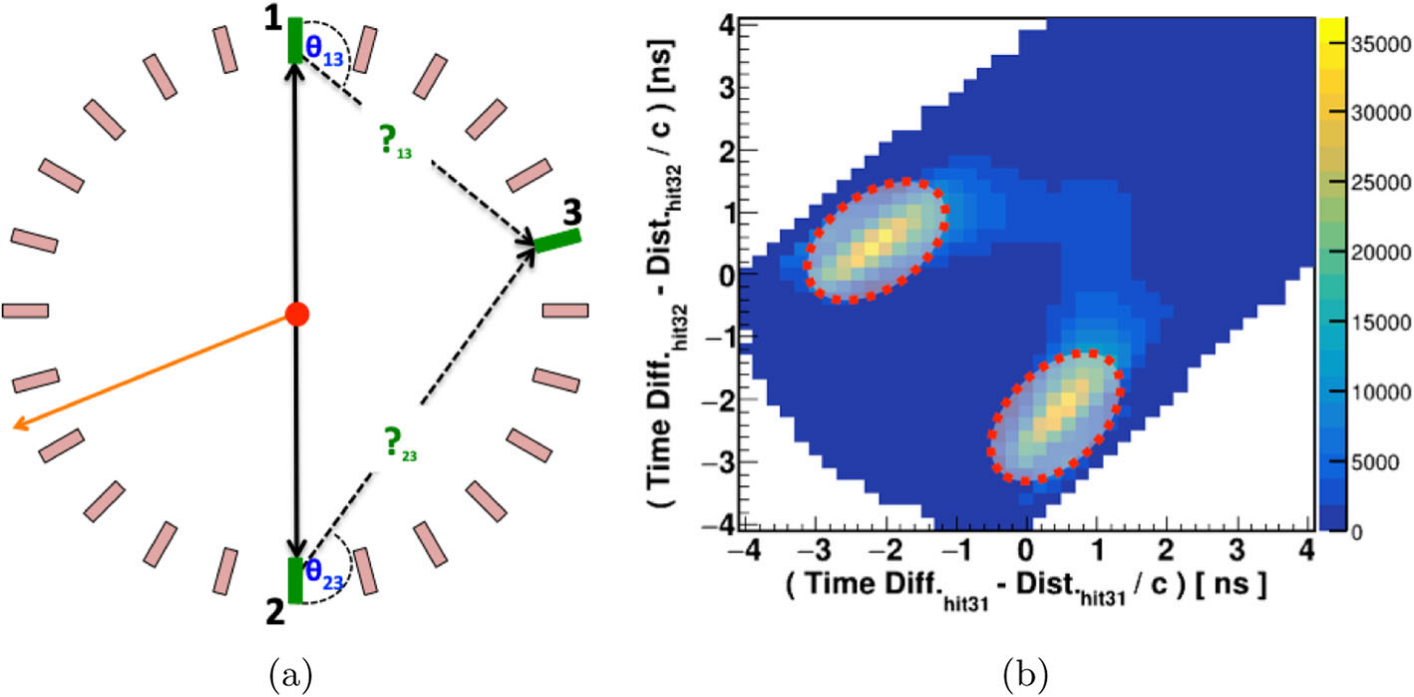
**a** Pictorial representation of the event selection based on the algorithm used to tag the 511 keV photons. **b** The encircled dotted red lines show the selected scattering events of back-to-back photons based on the scatter test **S**

The 511 keV photons are the outcome of *e*^+^*e*^−^ annihilation into the back-to-back direction to satisfy the momentum conservation and can easily be identified in Fig. 6d as a vertical line at *α* + *β* = 180^∘^. The selection of such events was done based on the condition that the sum of the two smallest angles (*α*+*β*) should be 180^∘^ within the uncertainty of 2^∘^. The pictorial presentation of J-PET with one layer is used to visualize the situation. In Fig. 7a, black lines represent the back-to-back photons labeled as 1 and 2 and the orange line shows the prompt photon. Scintillators with photon hits are shown as green rectangles. Hit 3 corresponds to the interaction of the scattered photon after hit 1 or hit 2. The exact assignment of the scattering photons to its primary one is important for the proper estimation of scattering angle (Fig. 7b) which is essential for the determination of the energy deposition. For this purpose, a scatter test **S** is devised which estimates the time difference between the measured time (time difference between two hits) and calculated time (distance between hit-positions divided by the speed of light). The **S** test was applied event-wise assuming both possibilities that the third hit might belong to the scattering photon after hit 1 or hit 2. The results are shown in Fig. 7b. In case of the proper assignment, the value of *S* should be close to zero. For the final analysis, events from the areas encircled by red-dotted lines were taken into account. With the known incident photon’s energy (511 keV) and its scattering angle, the deposited energy is calculated in response to the initial hit. Thus, a one-to-one correspondence of energy deposition by 511 keV photons and TOT values on event-wise bases is extracted. In studying 511 keV photons, the relationship can be established only up to 340 keV of energy deposition (the Compton edge for 511 keV photon). So, in order to extend the relationship for higher energy deposition values, the studies with the prompt photons with energy 1275 keV were also performed. The selection criterion for the prompt gamma is explained in the next sub-section.

### Identification of 1275 keV photons and their scatterings

**Fig. 8:**
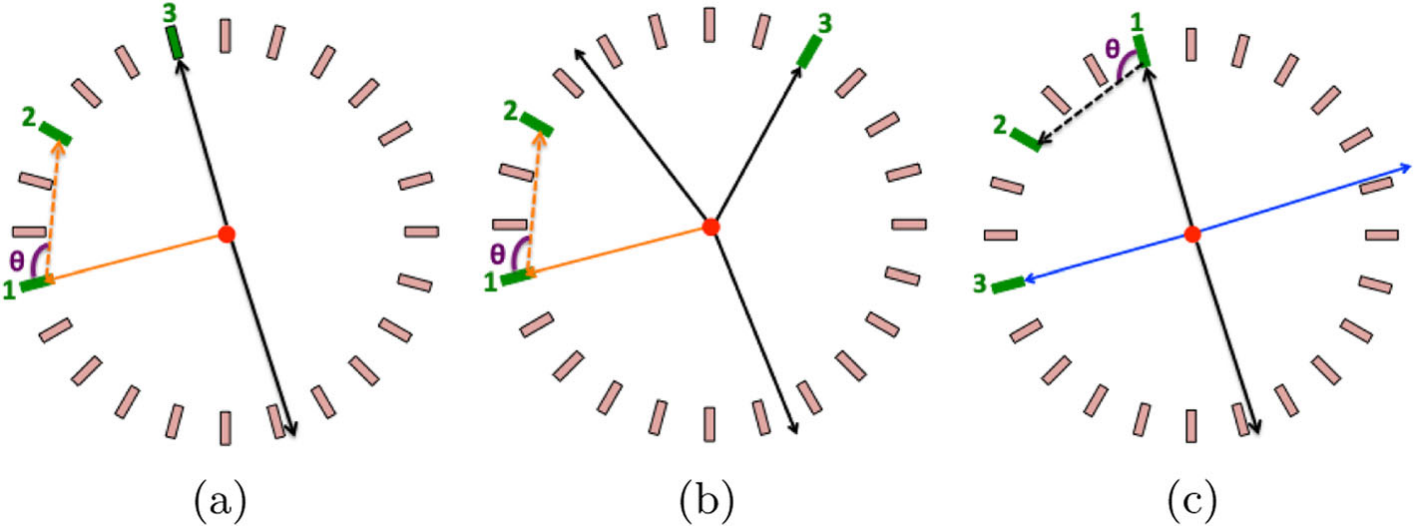
Three different cases in the events selection for the 1275 keV photons (orange arrows) based on the delayed decay of ortho-positronium atoms: **a** The first case shows the two annihilation photons (black arrows) from the decay of para-positronium (or ortho-positronium pick-off). **b** The photons originating from the decay of ortho-positronium state (three black arrows). **c** A possible source of background in the adopted procedure for the selection of prompt photon due to the accidental coincidences. The black and blue solid lines presenting the back-to-back annihilation photons might originate from two different decays

**Fig. 9:**
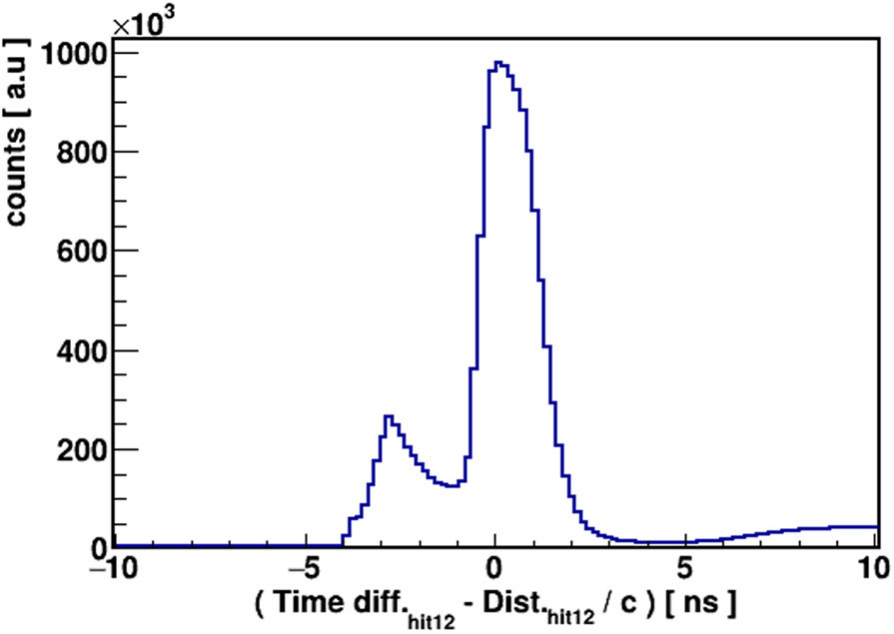
Results of the **S**-test calculated between hit 1 and hit 2 in event-wise manner. For the true scattering of prompt photons, the value of the S test is chosen between −0.5 and 0.75 ns. Small peak around *S* = −3 ns is mainly due to the contribution from back-to-back annihilation photons. A slope starting at 5 ns is because of the time difference constraint between hit 1 and hit 3 (> 10 ns)

The selection of prompt and its corresponding scatter photon without using an explicit cut on TOT values is not as straightforward as in the case of annihilation photons. As the first step in the selection procedure, in order to suppress events with back-to-back 511 photons, we applied cuts based on the angular correlation between the hits (Fig. 6d) such that we are not considering those events when the sum of two smallest angles lies in interval 165–185 ^∘^. In the next step, hits are ordered according to the ascending time and only those events are selected for which the time interval between first and third hit is larger than 10 ns. The assumed analogy is as follows: hit 1 is considered to be the prompt photon, hit 2 is the scattered photon of the prompt, and hit 3 is assumed by one of the photons originating from pick-off (Fig. 8a) or direct from the decay of ortho-positronium atoms (Fig. 8b). Figure 8c shows the possible background in the adopted selection criterion. The time difference (*Δt*) between the first and third hit is used as a key parameter. It is chosen to be much larger than the possible time difference between the scatterings of photon in the tomograph. For the present analysis, the value for *Δ*t was fixed in the time interval (10–100 ns). For estimating the scattering angles of prompt gamma, the scattered test was applied between first and second hit. The histogram used for the scatter test is shown in Fig. 9. The acceptance time window for the true scattering of the *S* test is chosen between − 0.5 and 0.75 ns.

## Theoretical model

The energy deposition by a photon inside a plastic scintillator is a function of scattering angle. Furthermore, the shape and amplitude of the signal pulse vary depending on the properties of scintillators (solvent, solute, and wavelength shifter) [50, 51]. The time distribution spectra of the emitted light in plastic scintillators EJ-230 (Eljen Technology) due to an incoming photon interaction at time *Θ* can be estimated by the following equation [3, 50, 51]:

3 $$ \hspace{1.5cm} f(t|\Theta) = K \int_{\Theta}^{t}{(e^{-\frac{t-\tau}{t_{d}}}-e^{-\frac{t-\tau}{t_{r}}}) \cdot e^{-\frac{(\tau-\Theta-2.5\sigma)^{2}}{2\sigma^{2}}} d\tau}   $$

**Fig. 10:**
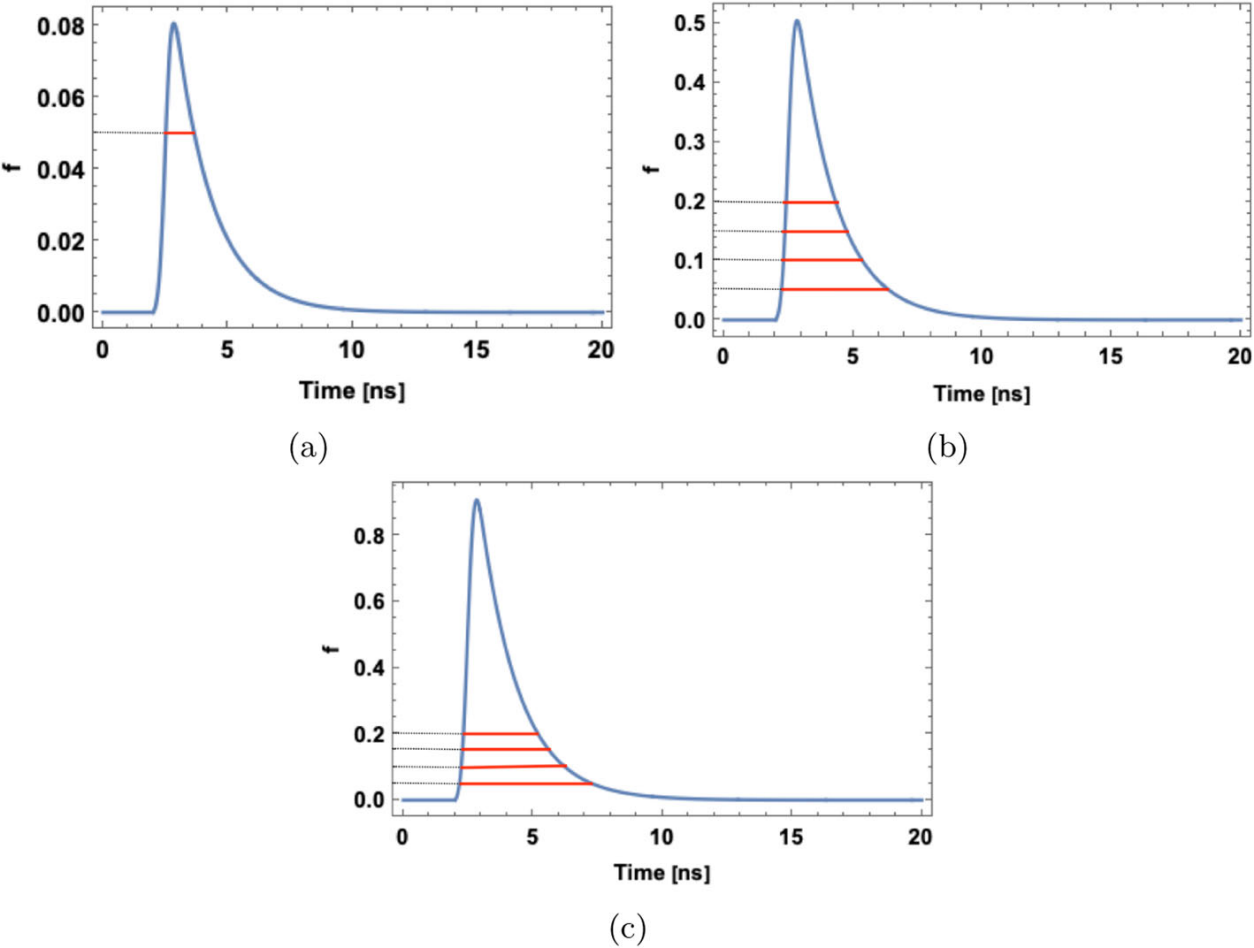
Time distribution spectra of emitted light is calculated by using Eq. 3 for three cases representing signals corresponding to the energy deposition of 80 keV (**a**), 500 keV (**b**), and 900 keV (**c**)

The equation is a convolution of Gaussian and exponential terms, where *σ* represents the rate of energy transfer to the primary solute, whereas *t*_*r*_ and *t*_*d*_ denote the average time of the energy transfer to the wavelength shifter and decay time of the final light emission, respectively [51]. *K* is the normalization factor to unity. At time *Θ* = 2 ns, t _*d*_ = 1.5 ns, t _*r*_ = 0.005 ns, and *σ* = 0.2 ns, Eq. 3 gives the time distribution spectra of emitted photons where the deposited energy is characterized by the amplitude of the signal (f). Figure 10 indicates three exemplary pulses calculated according to Eq. 3 normalized relatively to simulate signals corresponding to energy loss of 80 keV (Fig. 10a), 500 keV (Fig. 10b), and 900 keV (Fig. 10c).

Each signal pulse is probed at four thresholds (f) 0.05 (TOT_1_), 0.10 (TOT_2_), 0.15 (TOT_3_), and 0.20 (TOT_4_) shown by the red solid lines in Fig. 10. The total TOT value is calculated as TOT _*model*_ = N _0 ·_ TOT _*sum*_, where TOT _*sum*_ = TOT_1_ + TOT_2_ + TOT_3_ + TOT_4_ and N_0_ is a free parameter. The theoretical predictions will be compared with experimental results in the next section.

## Results

The motivation of this work is the elaboration of a method to estimate the energy loss by incident photons interacting with plastic scintillators used in the J-PET tomography scanner. The analysis is performed with the ^22^Na isotope because it is long lived (2.6 years half lifetime) and is emitting positrons annihilating to 511 keV photons as used in PET diagnostics. Since the aim of this study is to establish the relation between TOT and *Δ*E for the plastic scintillators, in order to avoid the biasness in identification of 511 keV (positron annihilation) and 1275 keV (deexcitation) photons, a new method of photon identification was developed which is independent of the TOT values. After selecting the photons of different energies, their scattering angles are estimated based on the hit characteristics. With information of incident energy of photon and its scattering direction, the energy transfer to the electrons inside the scintillator can be calculated using the Compton scattering formula (Eq. 2).

**Fig. 11:**
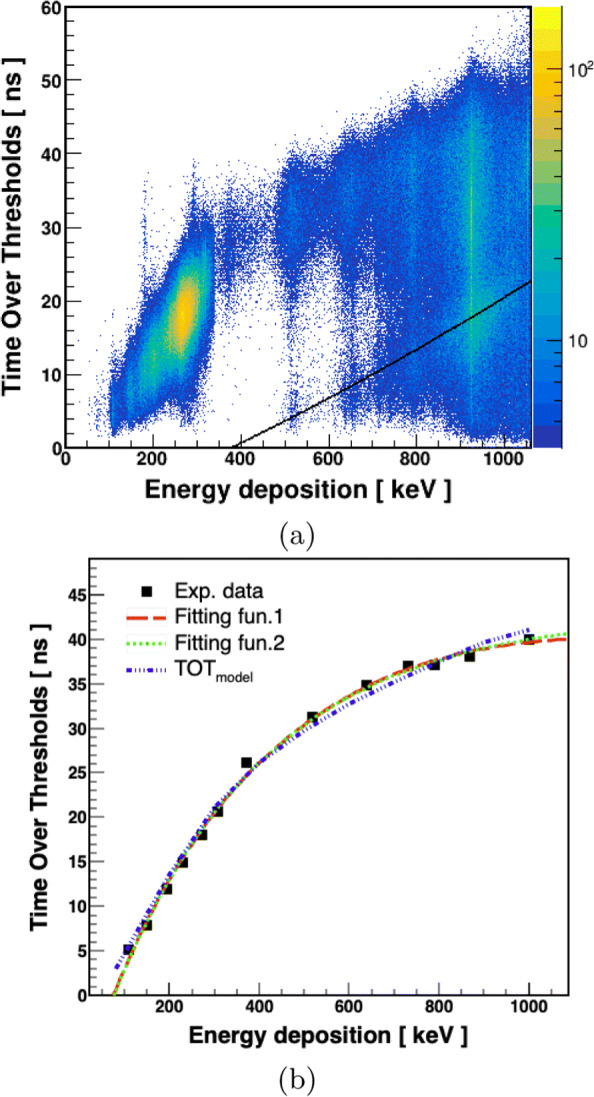
**a** 2-D spectrum of TOT versus energy deposition. **b** TOT vs energy deposition. Black rectangles correspond to the experimental data. The statistical errors in measuring the values are smaller than the size of symbols due to the large number of entries for the fitted TOT distributions. The red dashed line indicates result of the fit of the function: TOT = A0 + A1 * ln(E _*dep*_ + A2) + A3 * (ln(E _*dep*_ + A2))^2^, with A0 = -2322 ns, A1 = 632.1 ns/keV, A2 = 590.2 keV, and A3 = -42.29 ns/(keV)^2^. Green dotted line shows the result of another fitting function TOT = A0 ns - A1 * A2$^{E_{dep}}$ with three parameters where A0 = 42.96 ns, A1 = 53.43 ns, and A2 = 0.997 keV ^−1^. Blue dotted-dashed line represents the model predictions (the “Theoretical model” section) for the total TOT values at four fixed thresholds for the time distribution spectra calculated by using Eq. 3. In framework of J-PET, the light signals are collected on both sides of plastic scintillator as a measure of energy deposition, so in calculating the TOT _*model*_, we used twice the value of TOT _*sum*_ and the value of free parameter N_0_ is 1.3

In each event, hit characteristics are measured that comprise the TOT values and photon’s interaction time as well as spatial coordinates. For all selected hits, the relationship between measured TOT values and the estimated energy deposition is presented in Fig. 11. An enhancement in TOT values is observed with increasing energy depositions which is an expected behavior. However, some contributions are visible with large energy deposition but with smaller TOT values; this can be interpreted as wrongly tagged photons due to accidental coincidences, i.e., instead of 1275 keV photon, photons with lower incident energies were used to estimate the energy deposition for the scattering angles.(e.g., see the Fig. 8c). The accidental coincidence rate for the presented analysis is estimated ≈ 5%. The black line in Fig 11a is plotted by assuming that the wrongly tagged photons are of energy 511 keV. For the final relationship, the profile histograms of Fig. 11a for the most populated energy bins are selected. The TOT spectra for selected energy intervals fitted for a fixed range around the maximum of distribution. The mean values of the TOT distributions as a function of the center value of the energy interval are shown in Fig. 11b. The black rectangles are the experimental data. Errors in estimating the mean value of the TOT are within the size of the used symbols. It is shown that the used functions (see the caption of Fig. 11) are able to reproduce the data for quite a large range of energy deposition, i.e., up to 1000 keV. The experimental data is confronted also with a theoretical model (the “Theoretical model” section) which well describes the data.

## Conclusions

J-PET is the first PET scanner composed of plastic scintillators [3, 30]. Plastic scintillators are more than an order of magnitude less expensive than crystal scintillators. The time-over-threshold approach facilitates a compact, fast, and cost-effective signal readout [32, 42, 43]. In the framework of J-PET scanner, we adopt the TOT approach as a measure of the energy deposition in order to utilize the fast timing and low pile-up features of the plastics scintillators. To apply this method effectively, in comparison to the classical method of charge collection, it is important to precisely describe the non-linear relationship between the energy deposition of an initial photon and the measured TOT values. Here, we presented a method for determining this relation which we showed can be efficiently applied to the J-PET tomograph by collecting the data with a ^22^Na source covered with the porous material characterized with the long lifetime of positronium atoms [29]. The identification of an incident photon was based on the angular correlation between the three hits and the lifetime of the metastable positronium atoms while the scattered photon was identified and associated to the initial photon based on the correlation among the registration time and distance between the interaction points. Using a ^22^Na source emitting 1275 keV prompt and 511 keV annihilation photons, the TOT versus energy loss relationship up to about 1000 keV was established. The proposed functions fits the experimental data well and can be used as a standalone functions for the energy loss calibration of the plastic scintillators used in the J-PET scanner. Furthermore, we also introduced a theoretical model following a simple algorithm able to reproduce the experimental data which may be used in other experimental facilities using the plastic scintillators.

## Data Availability

The data that support the findings of this study are available from the corresponding author upon request.
